# A chromosome-scale genome assembly of *Castanopsis hystrix* provides new insights into the evolution and adaptation of Fagaceae species

**DOI:** 10.3389/fpls.2023.1174972

**Published:** 2023-04-25

**Authors:** Wei-Cheng Huang, Borong Liao, Hui Liu, Yi-Ye Liang, Xue-Yan Chen, Baosheng Wang, Hanhan Xia

**Affiliations:** ^1^ College of Horticulture and Landscape Architecture, Zhongkai University of Agriculture and Engineering, Guangzhou, China; ^2^ Key Laboratory of Plant Resources Conservation and Sustainable Utilization, South China Botanical Garden, Chinese Academy of Sciences, Guangzhou, China; ^3^ South China National Botanical Garden, Chinese Academy of Sciences (CAS), Guangzhou, China

**Keywords:** *Castanopsis hystrix*, cellulose synthase (CesA) gene, chromosome-scale genome assembly, comparative genomic analysis, gene family

## Abstract

Fagaceae species dominate forests and shrublands throughout the Northern Hemisphere, and have been used as models to investigate the processes and mechanisms of adaptation and speciation. Compared with the well-studied genus *Quercus*, genomic data is limited for the tropical-subtropical genus *Castanopsis*. *Castanopsis hystrix* is an ecologically and economically valuable species with a wide distribution in the evergreen broad-leaved forests of tropical-subtropical Asia. Here, we present a high-quality chromosome-scale reference genome of *C. hystrix*, obtained using a combination of Illumina and PacBio HiFi reads with Hi-C technology. The assembled genome size is 882.6 Mb with a contig N50 of 40.9 Mb and a BUSCO estimate of 99.5%, which are higher than those of recently published Fagaceae species. Genome annotation identified 37,750 protein-coding genes, of which 97.91% were functionally annotated. Repeat sequences constituted 50.95% of the genome and LTRs were the most abundant repetitive elements. Comparative genomic analysis revealed high genome synteny between *C. hystrix* and other Fagaceae species, despite the long divergence time between them. Considerable gene family expansion and contraction were detected in *Castanopsis* species. These expanded genes were involved in multiple important biological processes and molecular functions, which may have contributed to the adaptation of the genus to a tropical-subtropical climate. In summary, the genome assembly of *C. hystrix* provides important genomic resources for Fagaceae genomic research communities, and improves understanding of the adaptation and evolution of forest trees.

## 1 Introduction

The Fagaceae family includes nine genera and roughly 900 species, which dominate forests and shrublands throughout the Northern Hemisphere (Oh and Manos, 2008; Petit et al., 2013). The three largest genera, *Quercus* (about 450 species), *Lithocarpus* (about 300 species), and *Castanopsis* (about 120 species) rapidly diverged after the Cretaceous-Paleogene boundary (K-Pg) (Zhou et al., 2022b) and currently occupy various habitats (Petit et al., 2013; Cannon et al., 2018). *Quercus* species are the dominant tree species of temperate forests in Eurasia and North American, while *Castanopsis* and *Lithocarpus* are mainly found in the tropical-subtropical evergreen forests of East and Southeast Asia (Petit et al., 2013; Cannon et al., 2018). Fagaceae species have been widely used as models of ecological and evolutionary genomic studies for the investigation of the processes and mechanisms of adaptation and speciation (Petit et al., 2013; Cavender-Bares, 2019; Kremer and Hipp, 2020). To date, more than 10 genomes of *Quercus* species have been assembled (**Table 1**), and the genomes of a dozen to one hundred individual oaks, such as those of *Q. acutissima* (Fu et al., 2022; Yuan et al., 2023), *Q. dentata* (Zhou et al., 2022a), *Q. petraea* (Leroy et al., 2020) and *Q. variabilis* (Liang et al., 2022) have been re-sequenced. By contrast, there is only a limited amount of genomic data available for the genus *Castanopsis*, and only one genome assembly (*C. tibetana*) is available for this genus (Sun et al., 2022). Molecular markers have been used to investigate the genetic diversity and evolutionary history of *Castanopsis* species (Shi et al., 2011; Li et al., 2014; Sun et al., 2014; Sun et al., 2016; Jiang et al., 2020; Li et al., 2022). However, our knowledge of the evolution of those species is incomplete or possibly biased due to a lack of sufficient genomic data. The availability of whole genome-wide data would provide an unprecedented opportunity for acquiring a deeper understanding of the adaptation and evolution of the genus *Castanopsis*, and would expand Fagaceae genome resources for comparative analysis.

**Table 1:** Comparisons of genome assembly quality among 12 Fagaceae species.

Species	Sequencing platform	Genome size (Mb)	Percentage of scaffolds anchored to pseud-chromosome	Contig N50 (Mb)	Number of contigs	Scaffold N50 (Mb)	Number of scaffolds	BUSCOs (%)	No. of protein-coding genes	Average gene length (bp)	Percentage of repetitive sequences	Reference
*Castanopsis hystrix*	Illumina, Pacbio, Hi-C	882.69	98.07%	40.95	211	75.63	172	99.50%	37,750	4,819	50.95%	This study
*Castanopsis tibetana*	Illumina, ONT, Hi-C	878.64	98.67%	3.33	477	76.69	37	92.95%	40,937	4,857	54.30%	Sun et al. (2022)
*Castanea crenata*	Illumina, ONT, Hi-C	718.30	99.72%	6.36	206	NA	NA	97.60%	46,744	3,880	58.78%	Wang et al. (2022a)
*Castanea mollissima*	Illumina, PacBio, Hi-C	688.98	99.75%	2.83	671	57.34	112	92.44%	33,638	NA	53.24%	Wang et al. (2020)
*Quercus acutissima*	Illumina, PacBio, 10x Genomics	758.00	99.00%	1.44	770	2.89	388	90.50%	31,490	5,145	48.00%	Fu et al. (2022)
*Quercus gilva*	Illumina, PacBio, Hi-C	889.71	96.54%	28.32	773	70.35	515	98.60%	36,442	3,724	57.57%	Zhou et al. (2022c)
*Quercus lobata*	Illumina, PacBio, Hi-C	847.00	96.00%	1.90	NA	75.00	2014	95.00%	39,373	6,575	54.40%	Sork et al. (2022)
*Quercus mongolica*	Illumina, PacBio, Hi-C	809.84	95.65%	2.64	645	66.74	330	94.45%	36,553	6,085	53.75%	Ai et al. (2022)
*Quercus robur*	Illumina, Roche 454	789.35	96.00%	0.07	22,615	1.35	1409	91%	25,808	2,907	54.30%	Plomion et al. (2018)
*Quercus suber*	Illumina	953.00	Scaffold-level	0.08	36,760	0.50	23,344	95%	79,752	NA	51.60%	Ramos et al. (2018)
*Quercus variabilis*	DNBSEQ, PacBio, Hi-C	796.30	98.80	26.04	327	64.86	245	98%	32,466	5,272	67.60%	Han et al. (2022)
*Fagus sylvatica*	Illumina, PacBio, Hi-C	540.30	99.09%	0.14	6,650	46.56	167	97.40%	63,736	3,919	59.09%	Mishra et al. (2022)

NA, not reported in original paper.

*Castanopsis hystrix* (2n=2x=24) is one of the most important and dominant species of the tropical-subtropical evergreen forests of Asia (Li, 1996). In China, *C. hystri*x is naturally distributed in mixed and secondary forests, and its distribution extends from Nanling Mountain to Hainan Island and from Taiwan to south Tibet (Huang et al., 1999). *C. hystri*x is an ecologically and economically valuable species, and its forests play critical roles in water and soil conservation, disaster prevention, biodiversity, and the global carbon budget (Huang et al., 2015; You et al., 2018; Liang et al., 2019; Zhang et al., 2019a). *C. hystri*x is also a source of well-textured heartwood, which is widely used in furniture, construction, and shipbuilding, and it also produces seeds that can be used to extract tanning agents and starch (Chen et al., 1993; Chang et al., 1995). Due to the overexploitation of natural forests, the once widespread *C. hystrix* populations have been greatly diminished and fragmented (Zhao et al., 2020). High-quality genomic data are essential for assessing the patterns of genetic diversity, tracking the evolutionary history, and developing effective and efficient conservation strategies for this plant species. To date, only plastid and nuclear SSR markers have been used to investigate differences in the genetic diversity and divergence of *C. hystrix* (Li et al., 2007; Li et al., 2022); however, information on its nuclear genome is still unavailable.

In this study, we assembled and annotated the first chromosome-scale high-quality genome of *C. hystrix* by integrating PacBio HiFi long-reads, Illumina short-reads, RNAseq, and Hi-C sequencing data. We performed comparative genomic analysis to explore the evolution of genes, gene families, and genomes of *C. hystrix* and related Fagaceae species. Our study provides new insights into the genome evolution of Fagaceae tree species and provides essential genomic resources for germplasm conservation and genetic improvement of *C. hystrix*.

## 2 Material and methods

### 2.1 Plant sampling and genome sequencing

Fresh leaves were collected from an adult *C. hystrix* tree growing in Guangdong Fenghuangshan Forest Park (23.22° N, 113.39° E) and immediately frozen in liquid nitrogen until further use. Total genomic DNA was isolated from leave tissues using a DNeasy Plant MiniKit (Qiagen, Germany). The DNA quality and concentration were assessed by agarose gel electrophoresis and the Qubit Fluorometer (Thermo Fisher Scientific, USA). To obtain whole genome sequencing data, three DNA libraries were constructed and sequenced. First, an Illumina library with insert size of ~350 bp was sequenced on an Illumina NovaSeq 6000 platform with 150 bp paired-end reads. Second, a 20 kb HiFi library was prepared using the SMRTbell Express Template Preparation kit 2.0 (Pacific Biosciences, USA), and then sequenced on the Pacbio Sequel II platform to produce long-reads. Finally, a Hi-C sequencing library was constructed and sequenced on an Illumina NovaSeq 6000 platform (paired-end 150 bp).

Leaves at three different development stages (bud, immature, and mature) were collected from the same tree used for genome sequencing. Total RNA was extracted from leave samples using an RNAprep Pure Plus Kit (Tiangen, China), and the quality of RNA was evaluated using a Nanodrop spectrophotometer (Thermo Fisher Scientific, USA) and an Agilent 5400 (Agilent Technologies, USA). Total RNAs isolated from different leave tissues were mixed in equal amounts. A synthesized complementary DNA (cDNA) library was sequenced on an Illumina NovaSeq 6000 platform (paired-end 150 bp).

### 2.2 Genome survey and *de novo* assembly

To predict genomic characteristics, k-mer analysis was performed based on Illumina paired-end reads. The 17 bp *K*-mers were counted using Jellyfish v2.2.7 (Marcais and Kingsford, 2011), and genome size, heterozygosity, and repetitive element content were predicted based on the k-mer count distribution using GenomeScope v2.0 (Vurture et al., 2017).

The *de novo* assembly of *C. hystrix* genome was conducted in three steps by integrating Illumina short-reads, PacBio HiFi long-reads, and Hi-C sequencing data. First, the PacBio HiFi reads were error-corrected using NextDenovo v2.4.0 (https://github.com/Nextomics/NextDenovo), and were then initially assembled using Hifiasm v0.15.4 (Cheng et al., 2022). Second, the draft assembly was polished using NextPolish v1.3.1 (Hu et al., 2020), and redundant contigs were filtered using Redundans pipeline (Pryszcz and Gabaldón, 2016). Finally, contigs were linked to 12 pseudo-chromosomes of *C. hystrix* using ALLHiC (Zhang et al., 2019b) and Juicebox (Durand et al., 2016) based on Hi-C data. The quality of the genome assembly was evaluated using BUSCO (Benchmarking Universal Single-Copy Orthologs) (Seppey et al., 2019).

### 2.3 Prediction of genes and repetitive elements

The repeat regions, protein-coding genes, and non-coding RNA (ncRNA) were annotated in the *C. hystrix* genome assembly. Tandem repeats were identified using Tandem Repeats Finder v4.09 (Price et al., 2005), and dispersed repeats were identified by integrating *de novo* and homology-based methods. Briefly, *de novo* prediction was performed using LTR_FINDER v1.0.6 (Xu and Wang, 2007), LTR_retriever v2.9.0 (Ou and Jiang, 2018), RepeatScout v1.0.5 (Price et al., 2005), and RepeatModeler v2.0.1 (Flynn et al., 2020). The homology-based approach was conducted using Repeatmasker v4.1.0 (Chen, 2004). The *C. hystrix* assembly was searched against the RepBase library (Jurka et al., 2005) to identify sequences that are similar to known repetitive elements.

To annotate protein-coding genes, we conducted *de novo*, homology-based and RNA-Seq-assisted predictions on the repeat-masked *C. hystrix* genome. For *de novo* gene annotation, coding regions of genes were predicted using Augustus v3.2.3 (Stanke et al., 2006), Geneid v1.4 (Blanco et al., 2007), Genescan v1.0 (Burge and Karlin, 1997), GlimmerHMM v3.04 (Majoros et al., 2004), and SNAP (Aylor et al., 2006). For homology-based prediction, protein sequences of *Castanea mollissima* (Wang et al., 2020), *Castanopsis tibetana* (Sun et al., 2022), *Fagus sylvatica* (Mishra et al., 2018), *Quercus lobata* (Sork et al., 2016), *Quercus robur* (Plomion et al., 2018), and *Quercus suber* (Ramos et al., 2018) were downloaded from Genbank and aligned with the *C. hystrix* genome using TblastN v2.2.26 (Altschul et al., 1990). By comparing the homologous genome sequences to the matched proteins, gene models were constructed using GeneWise v2.4.1 (Birney et al., 2004). For RNA-Seq-based auxiliary prediction, a *C. hystrix* transcriptome was assembled using Trinity v2.1.1 (Grabherr et al., 2011) and aligned to the *C. hystrix* genome assembly using Hisat v2.0.4 (Kim et al., 2015). After that, gene models were predicted using PASA v2.0.2 (Keilwagen et al., 2016). Gene models predicted by the three methods were integrated using EvidenceModeler v1.1.1 (Haas et al., 2008), resulting in a non-redundant gene set. The ncRNAs, including rRNAs, micro RNAs (miRNAs), and small nuclear RNAs (snRNAs) were identified by searching the genome assembly against the Rfam database (Griffiths-Jones et al., 2003) with default parameters using Infernal v1.1 (Nawrocki and Eddy, 2013). tRNAs were predicted using the program tRNAscan-se v2.0 (Chan et al., 2021).

To infer gene functions, protein sequences were compared with those in Kyoto Encyclopedia of Genes and Genomes (KEGG) (Kanehisa and Goto, 2000), non-redundant (NR), Gene Ontology (GO) (Ashburner et al., 2000), SwissProt (Boeckmann et al., 2003), InterPro (Hunter et al., 2009), and protein family (Pfam) (Finn et al., 2014) databases using Blastp (E-value cutoff of 1e^-5^). The motifs and domains were characterized using InterProScan v5.31 (Zdobnov and Apweiler, 2001) by searching against public databases, including ProDom, PRINTS, Pfam, SMRT, PANTHER, and PROSITE.

### 2.4 Gene family evolution analyses

To track the gene family evolution, we analyzed the protein sequences of *C. hystrix* generated in this study together with those of 10 other species representing major lineages of Fagaceae and eudicots. Proteins of these species were downloaded from public databases. These species included *C. tibetana* (https://db.cngb.org; Accession number: CNA0019678), *C. mollissima* (https://ngdc.cncb.ac.cn; Accession number: GWHANWH00000000), and *Oryza sativa* (https://phytozome-next.jgi.doe.gov/info/Osativa_v7_0). Other seven species were downloaded from National Center for Biotechnology Information (https://www.ncbi.nlm.nih.gov/), including *Fagus sylvatica* (GCA_907173295.1), *Juglans regia* (GCF_001411555.2), *Malus domestica* (GCA_002114115.1), *Prunus persica* (GCA_000346465.2), *Populus trichocarpa* (GCA_000002775.5), *Quercus robur* (GCA_932294415.1), *Vitis vinifera* (GCA_000003745.3). We identified orthologous genes using OrthoFinder v2.5.4 (Emms and Kelly, 2019), and then aligned gene coding regions using the package ParaAT v2.0 (Zhang et al., 2012). Single gene alignments were concatenated using seqkit v1.3 (Shen et al., 2016), and poorly aligned regions were excluded using Trimal v1.4 (Capella-Gutierrez et al., 2009). Then, a maximum likelihood (ML) tree was constructed based on the alignment of orthologous genes using IQ-TREE v2.1.2 (Nguyen et al., 2015), and dated using MCMCTree in the PAML v4.9j package (Yang, 2007). Two fossil calibrations were used to constrain the age of nodes. The first split within the Fagaceae family (genus *Fagus* vs. the rest of the genera) was constrained to 82–81 million years ago (Mya) (Grímsson et al., 2016), and the divergence time between genera *Castanopsis* and *Castanea* was restricted to 52.2 Mya (Wilf et al., 2019). Based on the dated phylogenetic tree, the expansion and contraction of gene families were inferred using CAFÉ v4.2.1 (De Bie et al., 2006).

### 2.5 Genome synteny and whole genome duplication analyses

To investigate the syntenic relationship between *C. hystrix* and relative species, proteins of *C. mollissima* (Wang et al., 2020) and *C. tibetana* (Sun et al., 2022) were downloaded from Genbank and compared with the genome of *C. hystrix* using Blastp (E-value cutoff of 1e^-5^). Collinear blocks were inferred using MCScanX (Wang et al., 2012) and visualized in JCVI v1.2.20 (Tang et al., 2008). The times of whole genome duplication (WGD) events were inferred from the synonymous substitution rates (*Ks*) between paralogous and orthologous gene pairs. The *Ks* of gene pairs was calculated using the Nei-Gojobori algorithm as implemented in MCScanX.

### 2.6 Long terminal repeat retrotransposons analysis

To investigate the evolution of LTRs in *C. hystrix* and relative species, we identified LTRs in four Fagaceae species (*C. mollissima*, *C. tibetana*, *Q. robur*, and *F. sylvatica*) following the same procedure used for *C. hystrix* (see above). For full-length LTRs, the reverse transcriptase (RT) domains were identified using TEsorter v1.4.5 (Zhang et al., 2022), and were then aligned using MAFFT v7.475 (Katoh et al., 2002) with default parameters. The phylogenetic trees of LTRs were constructed based on the alignment of RT domains using FastTree v2.1.10 (Price et al., 2009). To estimate the insertion times (*T*) of full-length LTRs, the Kimura two-parameter distance (*K*) of each LTR-RT pair was calculated and converted to the insertion time using the formula *T* = *K*/2μ, where the substitution rate (μ) was estimated using the baseml program in the PAML package.

### 2.7 Evolutionary analysis of the CesA gene family

Hard and well-textured heartwood are typical features of *C. hystri*x trees (Watanabe et al., 2014). Cell wall and lignin metabolic pathway genes are essential for wood formation. The cellulose synthase (CesA) gene family is involved in primary cell wall formation and cellulose synthase is considered the most important enzyme in the synthesis of cellulose microfibrils in plant cells (Kumar and Turner, 2015; Wang et al., 2022b). Hence, we conducted genome-wide characterization of the CesA family in *C. hystri*x and three relative Fagaceae species (*C. mollissima*, *C. tibetana* and *Q. robur*). The CesA genes in each species were identified using two methods. First, CesA protein sequences of *A. thaliana* (Persson et al., 2007) and *O. sativa* (Hazen et al., 2002) were blasted against the genomes of *C. hystrix*, *C. mollissima*, *C. tibetana*, and *Q. robur*, and homologous genes with an E-value cutoff of 1e^-10^ were identified. Second, two DNA-binding domains (PF03552 and PF00535) from Pfam (https://pfam.xfam.org/) were searched against protein sequences of Fagaceae species using HMMER v3.3.2 (Finn et al., 2011). The unions identified by both methods were considered to be common elements. To verify the reliability of the intersected results, we analyzed the completeness of CesA gene domains using Pfam and the conserved domain database (CDD, https://www.ncbi.nlm.nih.gov/cdd/). Then, the theoretical isoelectric points (PI) and molecular weights of CesA proteins were analyzed on the ExPASy website (https://web.expasy.org/compute_pi/).

For phylogenetic analysis, the amino acid sequences of each CesA member were aligned using MUSCLE v3.8 (Edgar, 2004), and phylogenetic trees were constructed using IQ-TREE with 1000 bootstraps and online visualization using iTOL (https://itol.embl.de/) (Letunic and Bork, 2019). To investigate in detail the classification of protein motifs, Multiple Em for Motif Elicitation (MEME) (http://memesuite.org/) was used to annotate the conserved motifs in these proteins. The maximum number of motifs was set to 10 and the motif width was set 10 to100 in MEME analysis. Blastp and MCScanX were used to identify syntenic blocks and duplication events with default parameters and visualization using TBtools (Chen et al., 2020).

## 3 Results

### 3.1 Genome assembly and assessment

**Figure 1:**
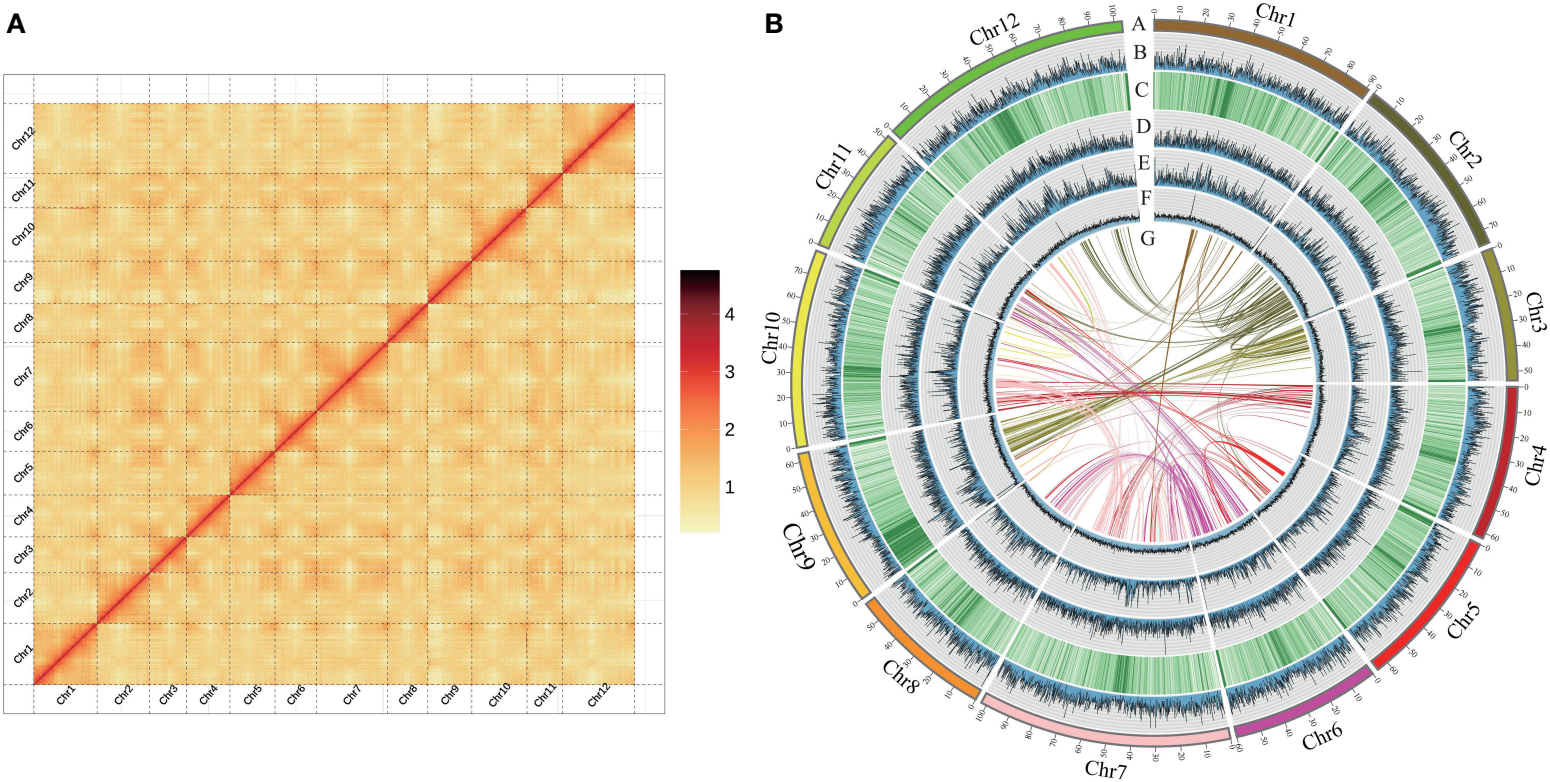
Features of *Castanopsis hystrix* genome. **(A)** Genome-wide analysis of chromatin interactions in the *C hystrix* genome based on Hi-C data. **(B)** The Synteny and distribution of genomic features. (A) The 12 pseudochromosomes; (B) gene density; (C–E) the density of total repeat sequences, Gypsy LTR-RTs, and Copia LTR-RTs; (F) histogram of GC content; (G) intragenomic collinearity. (B–F) were drawn in 100 kb overlapping sliding windows.

The *C. hystrix* genome was assembled by using integrated multiple sequencing and assembly technologies. Whole genome sequencing resulted in 52.92 Gb of Illumina short-reads (~59×), 28.14 Gb of PacBio HiFi long-reads (~31×), and 141.12 Gb of Hi-C data (~160×). An initial genome survey using k-mer analysis estimated that the genome size of *C. hystrix* is about 897.51 Mb and that it has a high level of heterozygosity of 1.26% and a repeat content of 57.38% (**Table S1**). Illumina short-reads, PacBio HiFi long-reads, and Hi-C sequencing data revealed that the assembled *C. hystrix* genome is 882.69 Mb, including 211 contigs and 172 scaffolds (**Table 1**). The contig N50 and scaffold N50 length are 40.95 Mb and 75.63 Mb, respectively. In total, 865.64 Mb (98.07%) of assembled sequences were mounted on 12 pseudo-chromosomes ranging from 51.51 Mb to 103.15 Mb (**Figure 1A**, **Table 1**). The heat map of Hi-C interactions shows that the genome assembly is intact and robust (**Figure 1B**).

The high accuracy and completeness of the *C. hystrix* genome assembly was supported by three analyses. First, joint analysis of GC content and sequencing depth revealed no obvious deviation in quality across the genome, suggesting the high quality of genome sequencing and assembly (**Figures 1**, **S1**). Second, approximately 97.66% of cleaned PacBio HiFi long-reads were successfully mapped to the genome, and more than 99% of the genome assembly had a coverage >10× (**Table S2**), suggesting that the genome assembly was accurate and complete. Finally, BUSCO analyses revealed that 99.5% of universal single-copy orthologs were present in the genome assembly (**Table 1**), indicating the high integrity of the genome assembly.

### 3.2 Genome annotation

A total of 449.72 Mb (50.95%) of the *C. hystrix* genome was annotated as repetitive sequences (**Tables 1**, **S3**). The most abundant repetitive elements were LTRs (374.50 Mb), followed by tandem repeats (47.64 Mb), long interspersed nuclear elements (LINEs; 18.08 Mb), DNA transposons (12.90 Mb), and short interspersed nuclear elements (SINEs; 16,791 bp) (**Table S3**).

By integrating *de novo*, homology-based, and RNA-Seq-assisted predictions, a total of 37,750 protein-coding genes were predicted in the *C. hystrix* genome (**Tables 1**, **S4**). The average lengths of coding sequences (CDSs), exons and introns are 1,067 bp, 244 bp and 1,112 bp, respectively (**Table S4**). By comparing the predicted gene set with six public databases, 36,962 (97.91%) of the total predicted genes were functionally annotated (**Table S5**). Non-coding RNA annotation identified 922 miRNAs, 741 tRNAs, 8,971 rRNAs, and 665 snRNAs in *C. hystrix* (**Table S6**).

### 3.3 Gene family evolution in *C. hystrix*

**Figure 2:**
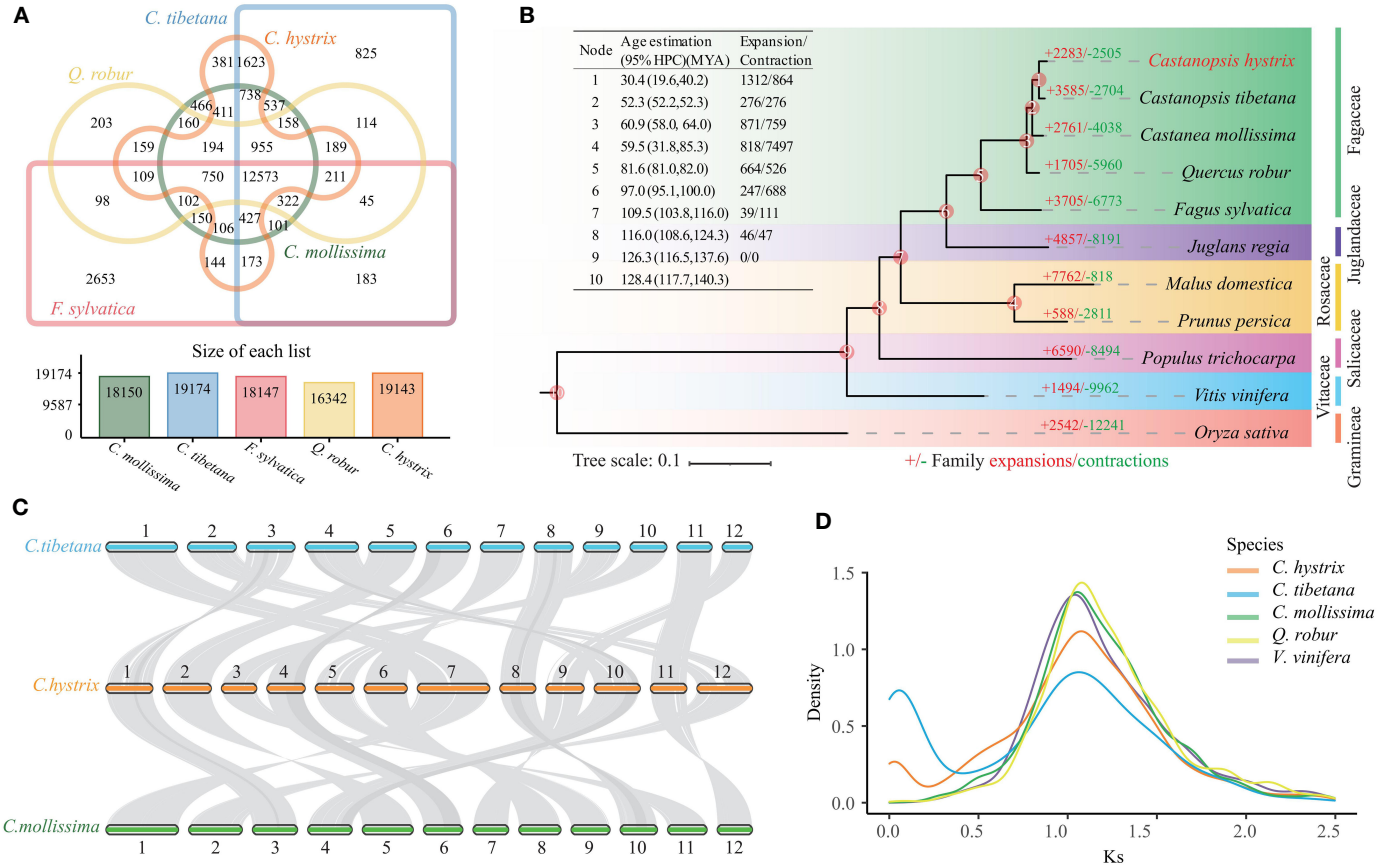
Genomic evolutionary and comparative genomic analyses. **(A)** Shared and unique gene families in *C hystrix*, *C tibetana*, *C mollissima*, *Q. robur*, and *F sylvatica*. **(B)** Phylogenomic tree and expansion and contraction of gene families among *C hystrix* and 10 other species. Numbers in red (+) and green (−) show the number of expanded and contracted gene families, respectively. **(C)** The synteny blocks between *C hystrix*, *C tibetana*, and *C mollissima.* Syntenic blocks were connected by grey lines. **(D)** The synonymous substitution rates (Ks) distributions of paralogous genes.

To explore the evolutionary history of the *C. hystrix* gene family, we clustered 36,448 (96.6%) annotated genes into 19,143 gene families. Among these, 12,573 gene families were shared with those of four other studied Fagaceae species (**Figure 2A**), and 299 families (1,043 genes) were unique to *C. hystrix*. Functional enrichment analysis showed that unique genes of *C. hystrix* were significantly enriched in 10 KEGG pathways and 115 GO terms, including Fatty acid biosynthesis, Porphyrin and chlorophyll metabolism, malate transport, and polynucleotide adenylyltransferase activity (**Table S7**; **Figure S2**).

A phylogenetic tree constructed using 556 single-copy orthologs among *C. hystrix* and other 10 angiosperms revealed that two *Castanopsis* species (*C. hystrix* and *C. tibetana*) were grouped together, and these two species are sister to a *Castanea* species (*C. mollissima*) (**Figure 2B**). Calibration of the phylogenetic tree using two Fagaceae fossil records showed that the divergence time between *C. hystrix* and *C. tibetana* is 30.4 Mya (95% HPD: 19.6–40.2 Mya) (**Figures 2B**, **S3**). The close phylogenetic relationships between *Castanopsis* and *Castanea* species were supported by the high genome synteny and colinearity (**Figure 2C**).

Based on the clustered gene families and dated phylogenetic tree, CAFÉ analyses detected 2283 expanded gene families and 2505 contracted gene families in *C. hystrix* (**Figure 2B**; **Tables S8**). Among these, 202 expanded and 62 contracted gene families were statistically significant (*P* < 0.01; **Table S8**). The 202 expanded gene families were enriched in 7 KEGG pathways and 36 GO terms, such as “Arginine and proline metabolism”, “Phenylalanine metabolism”, “Fatty acid degradation”, and “Trehalose biosynthetic process” (**Table S9**; **Figure S2**). The 62 contracted gene families were primarily enriched in KEGG pathway processes “Sesquiterpenoid and triterpenoid biosynthesis”, “Plant-pathogen interaction”, and “MAPK signaling” (**Table S9**). A search of *C. hystrix* expanded genes families against PlantTFDB (http://planttfdb.gao-lab.org/) revealed that 29 genes were categorized into four transcription factors (TFs) families (FAR1, B3, bHLH, and NAC). Among these, 23 genes belong to the FAR1 family, and the other six genes belong to B3 (one gene), bHLH (two genes), and NAC (three genes) families (**Table S10**). We also found that 17 and 16 gene families significantly expanded and contracted, respectively, in the most common ancestor of *C. hystrix* and *C. tibetana*. Functional enrichment analysis revealed that the 17 expanded gene families were overrepresented in 11 KEGG pathways and 8 GO terms, including “Fatty acid degradation”, “Plant-pathogen interaction” and “RNA-DNA hybrid ribonuclease activity” (**Table S9**). The 16 contracted gene families were enriched in six KEGG pathways and four GO terms (**Table S9**).

### 3.4 WGD in *C. hystrix*

Comparative genomic analyses were performed to discern the number of WGD events in *C. hystrix*. A total of 65 syntenic blocks (2,442 collinear genes) with sizes ranging from 11 to 48 gene pairs were detected in *C. hystrix*, accounting for 6.47% of the total gene set. The number of collinear genes in *C. hystrix* was close to those of other Fagaceae species (2484–2673 genes; 6.53%–7.71% of the total gene set) but lower than that in *V. vinifera* (3297 genes; 12.85% of the total gene set) (**Table S11**). The *Ks* values of paralogous and orthologous gene pairs showed that all four Fagaceae species and *V. vinifera* shared a *Ks* peak of approximately 1.08 units (**Figure 2D**), most likely representing the triplication event (γ) shared by all eudicots (Murat et al., 2015). Synteny analysis revealed a 1:1 syntenic depth ratio for *C. hystrix* vs. Fagaceae species and a 2:2 syntenic depth ratio for *C. hystrix* vs. *V. vinifera* (**Figure S4**). These results suggested that no independent WGD events have occurred in *C. hystrix* and other Fagaceae species.

### 3.5 Expansion of LTRs in *C. hystrix*

**Figure 3:**
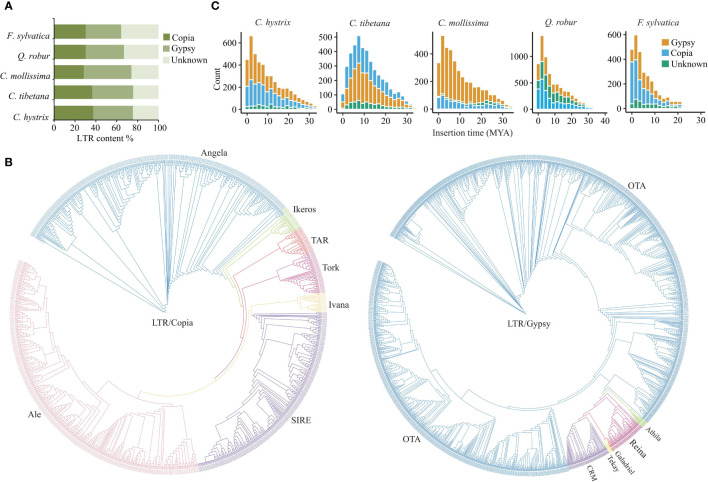
The features of LTR expansion in the Fagaceae genomes. **(A)** Comparison of LTR contents in *C hystrix* and 4 other species. **(B)** Neighbor-joining trees of Copia and Gypsy LTRs from *C hystrix*. **(C)** Insertion time estimates of full-length LTRs in five Fagaceae species.

Copia and Gypsy are the two most abundant LTR super families in *C. hystrix* and three other Fagaceae species. In *C. hystrix*, Copia- and Gypsy-type LTRs accounted for 37.49% and 38.04% of LTRs, respectively (**Figure 3A**; **Table S12**). The content of Copia- and Gypsy-type LTRs was slightly different among Fagaceae species (**Figure 3A**; **Table S12**), indicating independent expansion or elimination of repetitive elements. Phylogenetic analyses using RT domains of LTRs revealed that Copia-type elements were clustered into seven major groups, with Ale-type repeats forming the largest group (*N* = 355) followed by Angela (*N* = 320), SIRE (*N* = 235), Tork (*N* = 46), TAR (*N* = 29), Ikeros (*N* = 28), and Ivana (*N* = 21; **Figure 3B**). The Gypsy-type elements were grouped into six clades, and the OTA group accounted for 91% (1,658) of Gypsy members (**Figure 3B**). Full analyses with all Gypsy and Copia elements from the five Fagaceae species showed that the lineages of Copia and Gypsy were grouped according to their respective tribes, indicating different evolutionary relationships among LTR families (**Figure S5**). To further explore the details of LTR expansion, we estimated the insertion time of full-length LTRs. In *C. hystrix*, the insertion time peaks of both Copia- and Gypsy-type LTRs were found approximately at 2 Mya, while a more ancient amplification peak was found around 8 Mya in *C. tibetana* (**Figure 3C**). In other Fagaceae species, a significant burst of LTRs was detected at 1–3 Mya, but the extent of expansion varied among species and was also different between Copia- and Gypsy-type LTRs (**Figure 3C**).

### 3.6 Evolution of the CesA gene family

**Figure 4:**
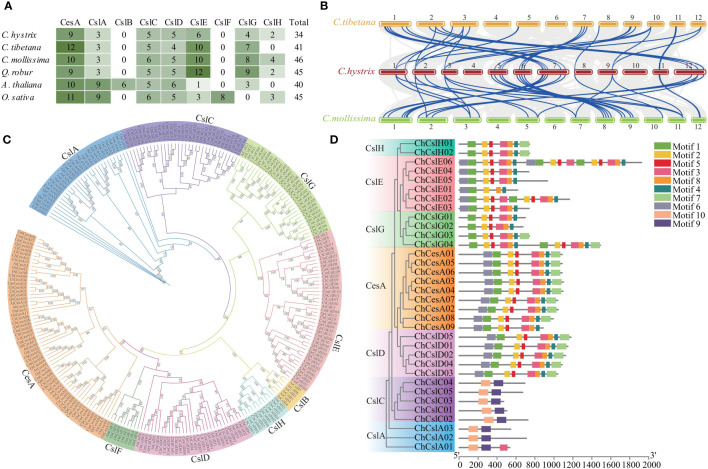
Identification and Evolution of CesA family in Fagaceae. **(A)** The heatmap shown a comparison of the numbers of CesA genes among four Fagaceae plants, *Arabidopsis thaliana*, and *Oryza sativa*. **(B)** Synteny analysis of CesA genes between *C hystrix*, *C tibetana*, and *C mollissima*. The blue lines highlight the syntenic CesA gene pairs. **(C)** Phylogenetic tree of CesA gene families in four Fagaceae plants, *A thaliana*, and *O. sativa*. **(D)** Phylogenetic relationships and architecture of the conserved protein motifs in 34 CesA genes from *C hystrix*.

Genome-wide characterization of the CesA family in *C. hystri*x identified 34 CesA-like genes (**Figure 4A**; **Table S13**). Phylogenetic analysis suggested that these genes could be divided into seven subfamilies (CesA, CslA–CslH) (**Figure 4C**; **Table S13**). Genes from the same subfamily showed similar protein domains and motif compositions, supporting their phylogenetic relationships (**Figures 4D**, **S6**). Similar numbers of CesA-like genes were found in three closely related Fagaceae species (41, 46, and 45 genes in *C. tibetana*, *C. mollissima*, and *Q. robur*, respectively) and two distinct related species, *A. thaliana* (40 genes) and *O. sativa* (45 genes) (**Figure 4A**; **Table S14**). However, Fagacea species showed different CesA subfamily content to that of *A. thaliana* and *O. sativa* (**Figure 4A**). For example, the number of CslE and CslG genes in Fagaceae species (6–12 and 4–9, respectively) was much higher than in *A. thaliana* (one and three, respectively) and *O. sativa* (three and none, respectively) (**Figure 4A**). Nine CslA genes were identified in *A. thaliana* and *O. sativa*, but only three CslA members were found in Fagaceae species (**Figure 4A**). In addition, the collinearity of CesA-like gene between *C. hystri*x and other Fagaceae species was clearly higher than those for *C. hystri*x vs. *A. thaliana* and *O. sativa* (**Figures 4B**, **S7**). An analysis of the distribution of CesA-like genes across the genome of *C. hystri*x revealed tandem duplication of 10 CesA genes (**Figure S8**).

## 4 Discussion

In this study, we generated a high-quality chromosome-scale assembly of *C. hystrix*. The assembled genome was approximately 882.6 Mb, of which more than 98% of the sequences were anchored to 12 pseudo-chromosomes ranging from 51.5 to 103.2 Mb in size. The contig N50 of the *C. hystrix* genome assembly was 40.95 Mb, which is higher than those of recently published Fagaceae species, such as *C. tibetana* (3.32 Mb) (Sun et al., 2022), *C. mollissima* (2.83 Mb) (Wang et al., 2020), *Castanea crenata* (6.36 Mb) (Wang et al., 2022a), *Quercus gilva* (28.32 Mb) (Zhou et al., 2022c), *Q. lobata* (1.90 Mb) (Sork et al., 2022), *Quercus variabilis* (26.04 Mb) (Han et al., 2022), and *F. sylvatica* (0.14 Mb) (Mishra et al., 2022). Genome assembly integrity, as assessed by BUSCO, reached 99.5% for *C. hystrix*, surpassing that of previously assembled Fagaceae genomes (90.5%–98.6%; **Table 1**). The high quality of the genome assembly can be mainly attributed to the successful implementation of new sequencing technologies, a statistical algorithm, and analytical approaches. Although gap-free T2T genomes are available in model species (Naish et al., 2021; Song et al., 2021), *de novo* genome assembly is still challenging for forest trees because of their large and complex genomes. Our genome assembly of *C. hystrix* is one of the most high-quality genomes of Fagaceae species ever reported.

Based on comparative genome analysis, we found high genome synteny between *C. hystrix* and *C. tibetana* and *C. mollissima*, although these species diverged more than 30 million years ago (Zhou et al., 2022b). We also found that *C. hystrix* and other investigated Fagaceae species did not experience WGD after the triplication event (γ) (Murat et al., 2015). These results are consistent with the previous hypothesis that ploidy level and genome structure are conserved among Fagaceae species, which may have facilitated the adaptive introgression between species (Chen et al., 2014; Cannon and Petit, 2020). Transposable elements (TEs) account for large parts of plant genomes, where they play an important role in evolution (Bennetzen and Wang, 2014; Akakpo et al., 2020). The proportion of the repetitive elements in the *C. hystrix* genome was 50.95%, similar to that reported for other Fagaceae, such as *C. tibetana* (54.30%) (Sun et al., 2022), *C. mollissima* (53.24%) (Wang et al., 2020), *Q. mongolica* (53.75%) (Ai et al., 2022), and *Q. variabilis* (26.04 Mb) (Han et al., 2022). Evolutionary analyses of LTRs showed that *C. hystrix* and relative Fagaceae species experienced a recent large-scale LTR burst, but the time and extent of LTR expansion varied between species and between LTR families, which may have influenced the structure and function of genomes and contributed to the adaptation and evolution of Fagaceae species.

Whole genome annotation and analysis revealed considerable gene family expansion and contraction in *C. hystrix* and relative species. These expanded and contracted gene families were involved in multiple important biological processes and molecular functions, providing valuable information for understanding the genetic basis of adaptation, evolution, and speciation in Fagaceae. For example, 17 gene families expanded in the most recent ancestor of *C*. *tibetana* and *C. hystrix*, and 202 gene families independently expanded in *C. hystrix*. Functional enrichment analysis suggested that the 17 expanded gene families were highly overrepresented in stress and defense-associated pathways, such as plant–pathogen interaction and Fatty acid degradation (Kindl, 1993; Goepfert and Poirier, 2007; Dodds and Rathjen, 2010; Chhajed et al., 2020). Fatty acid degradation is essential for seed development, seed germination, and post-germinative growth before the establishment of photosynthesis (Kindl, 1993; Goepfert and Poirier, 2007). In addition, expanded gene families in *C. hystrix* were enriched in the biological processes “Phenylpropanoids”, which influences plant responses to biotic and abiotic stimuli (La Camera et al., 2004; Vogt, 2010), and “Arginine and proline metabolism”, which plays key roles in nitrogen distribution and recycling in plants (Slocum, 2005; Rennenberg et al., 2010). Several expanded genes in *C. hystrix* are also members of the transcription factor family FAR1, which modulates phyA signaling (Lin et al., 2007) and regulates the balance between growth and defense under shade conditions (Liu et al., 2019). Therefore, the gene family expansions might have facilitated the adaptation of the genus *Castanopsis* to a tropical-subtropical climate, after they had diverged from their deciduous counterparts in cool-temperate areas. Furthermore, CslE/CslG genes of the CesA family exhibited expansion and tandem duplication in Fagaceae species. CesA genes are involved in the biosynthesis of various polysaccharide polymers, in particular hemicelluloses (Richmond and Somerville, 2000; Lerouxel et al., 2006). A recent study suggested that the expansion of the CesA family might have contributed to the formation of the high-density timbers that are characteristic of Dipterocarpaceae species (Wang et al., 2022b). Thus, we suspect that CesA gene expansion might be related to the development of the high-density woods of Fagaceae species. Taken together, these considerations suggest that gene family expansions might have played critical roles in the genetic, morphological, and physiological innovations of Fagaceae species.

In conclusion, we obtained the first chromosome-scale genome assembly of *C. hystrix* using a combination of multiple sequencing and assembly approaches. Genome-wide characterization and evolutionary analysis provided novel insights into the genome evolution and key regulatory pathways of wood formation in Fagaceae species. The *C. hystrix* genome assembly contains both high-quality reference sequences and important functional genes, which expands the genome resources for Fagaceae species and opens the possibility of conducting comparative and functional genomic studies of forest tree species.

## Data availability statement

The data presented in the study are deposited in the National Genomics Data Center (NGDC) database, BioProject accession number PRJCA015225.

## Author contributions

HX designed this study. BW and Y-YL collected samples. W-CH, BL, HL, and X-YC analyzed the data. HX, W-CH, and BL wrote the paper. All authors read and approved the final manuscript. All authors contributed to the article and approved the submitted version.
