# Supplementary material for: A chromosome-scale genome assembly of Castanopsis hystrix provides new insights into the evolution and adaptation of Fagaceae species
Source: Front Plant Sci. 2023 Apr 25;14:1174972. doi: 10.3389/fpls.2023.1174972 (PMC10197965; doi:10.3389/fpls.2023.1174972)
Supplement: Supplementary file 1 [file DataSheet_1.docx]

Supplementary Material

A chromosome-scale genome assembly of *Castanopsis hystrix* provides new insights into the evolution and adaptation of Fagaceae species

Wei-Cheng Huang^1,†^, Borong Liao^1,†^, Hui Liu^2,3^, Yi-Ye Liang^2,3^, Xue-Yan Chen^2,3^, Baosheng Wang^2,3^, Hanhan Xia^1*^

*** Correspondence:** Hanhan Xia, xiahanhan@zhku.edu.cn

# Supplementary Figures

**
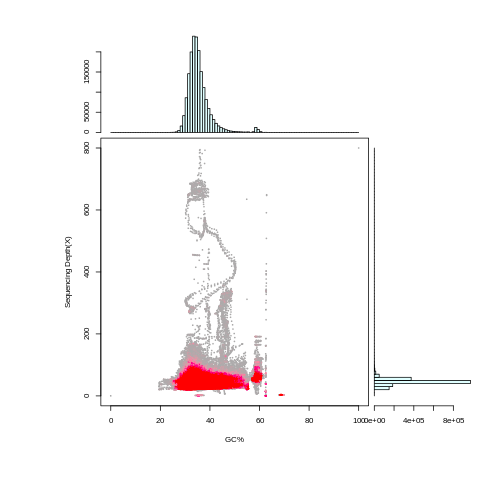
**

**Supplementary Figure 1.** Distribution of GC content and sequencing depth of the *C. hystrix* genome assembly.


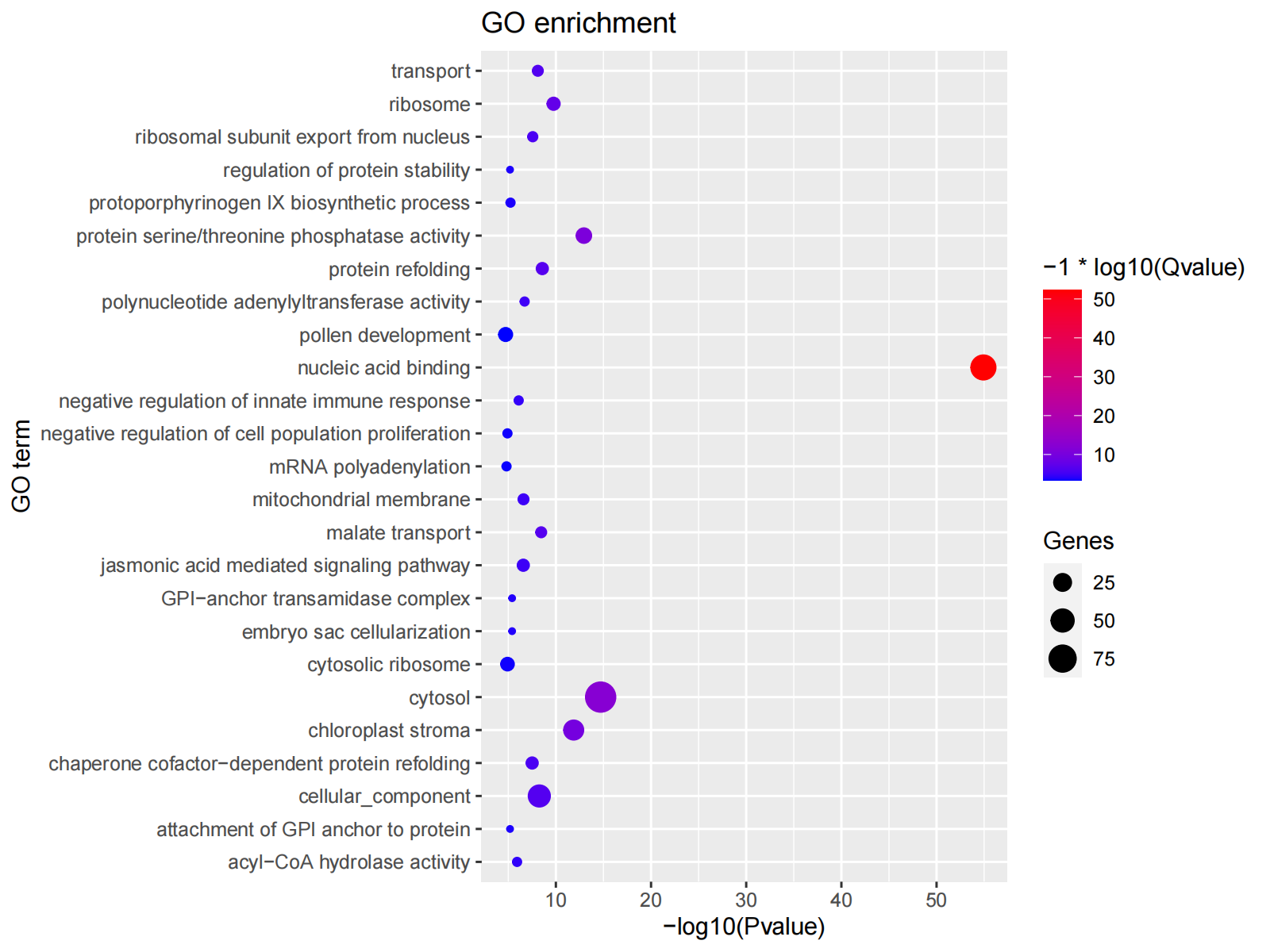

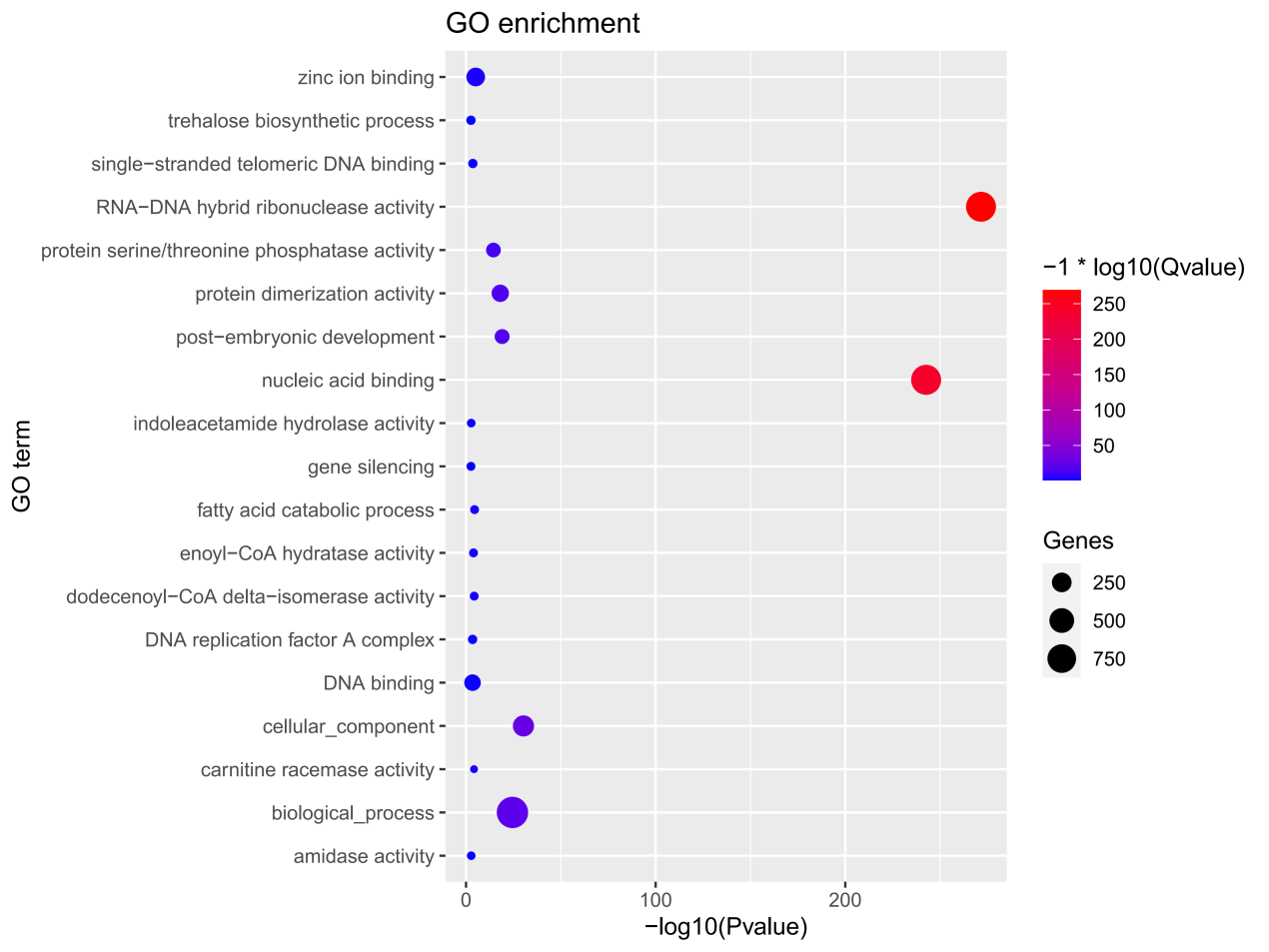


**a**

**b**

**Supplementary Figure 2.** GO term enrichment of unique gene families (a) and significantly expanded gene families (b) in the *C. hystrix* genome.

**
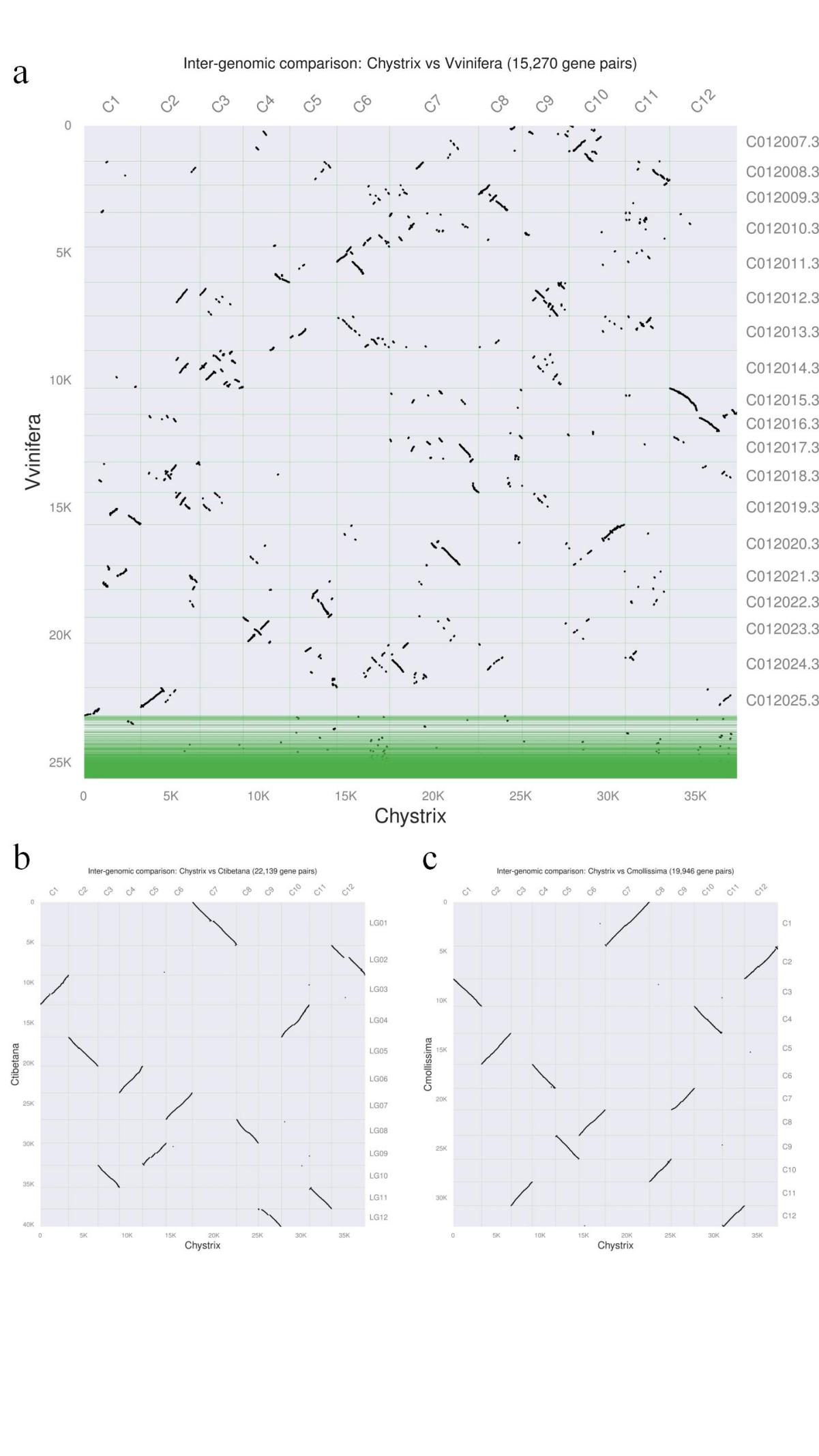
**

**Supplementary Figure 3.** Orthologous gene pairs in syntenic blocks between *C. hystrix* and related species. (a) syntenic gene pairs between *C. hystrix* and grape; (b) syntenic gene pairs between *C. hystrix* and *C. tibetana*; (c) syntenic gene pairs between *C. hystrix* and *C. mollissima*.

**
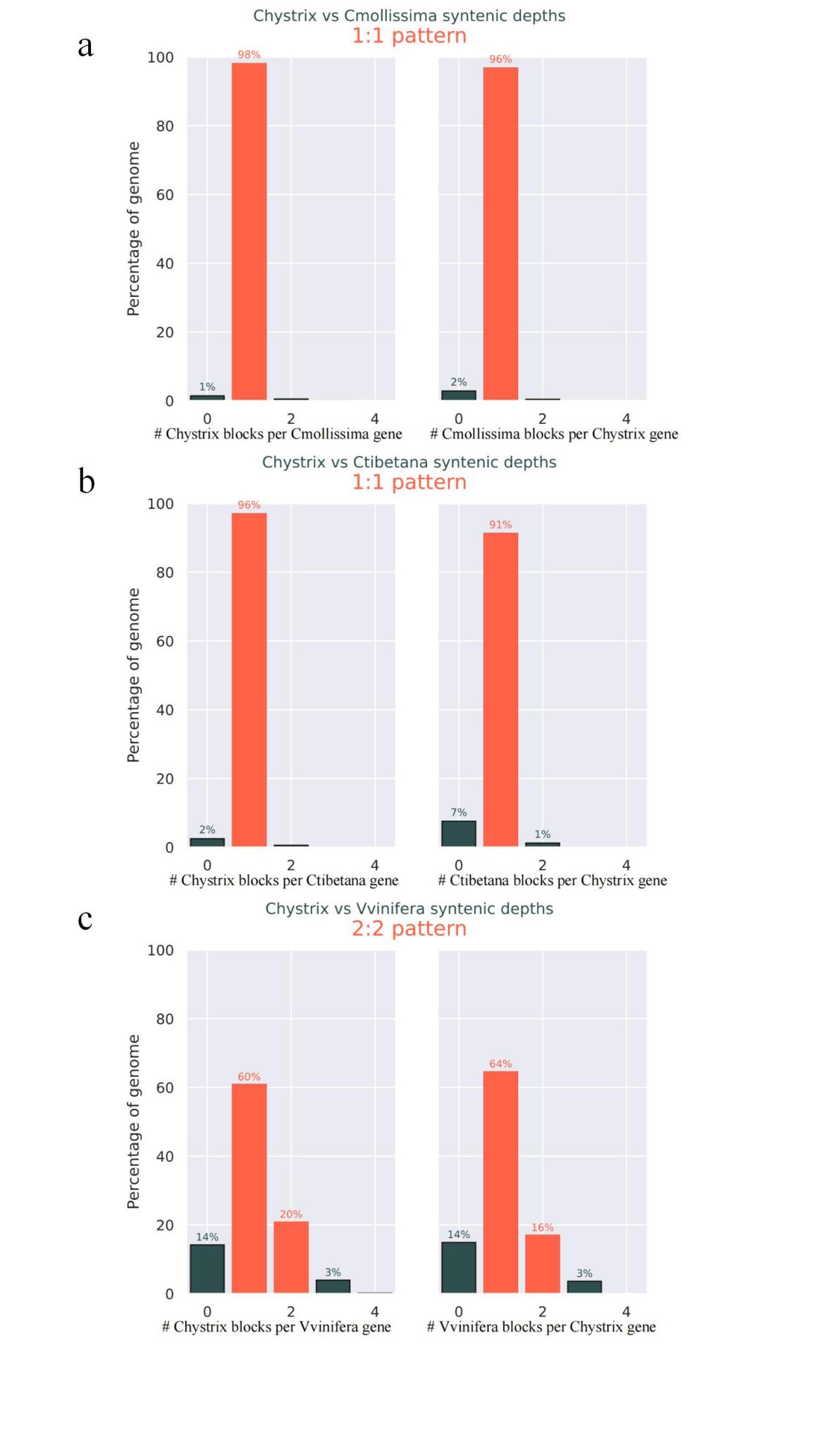
**

**Supplementary Figure 4.** The genome syntenic depth relationships between *C. hystrix* and two other Fagaceae species and grape. The species abbreviations are as follows: *Castanopsis hystrix* (Chystrix), *Castanea mollissima* (Cmollissima), *Castanopsis tibetana* (Ctibetana), and *Vitis vinifera* (Vvinifera).


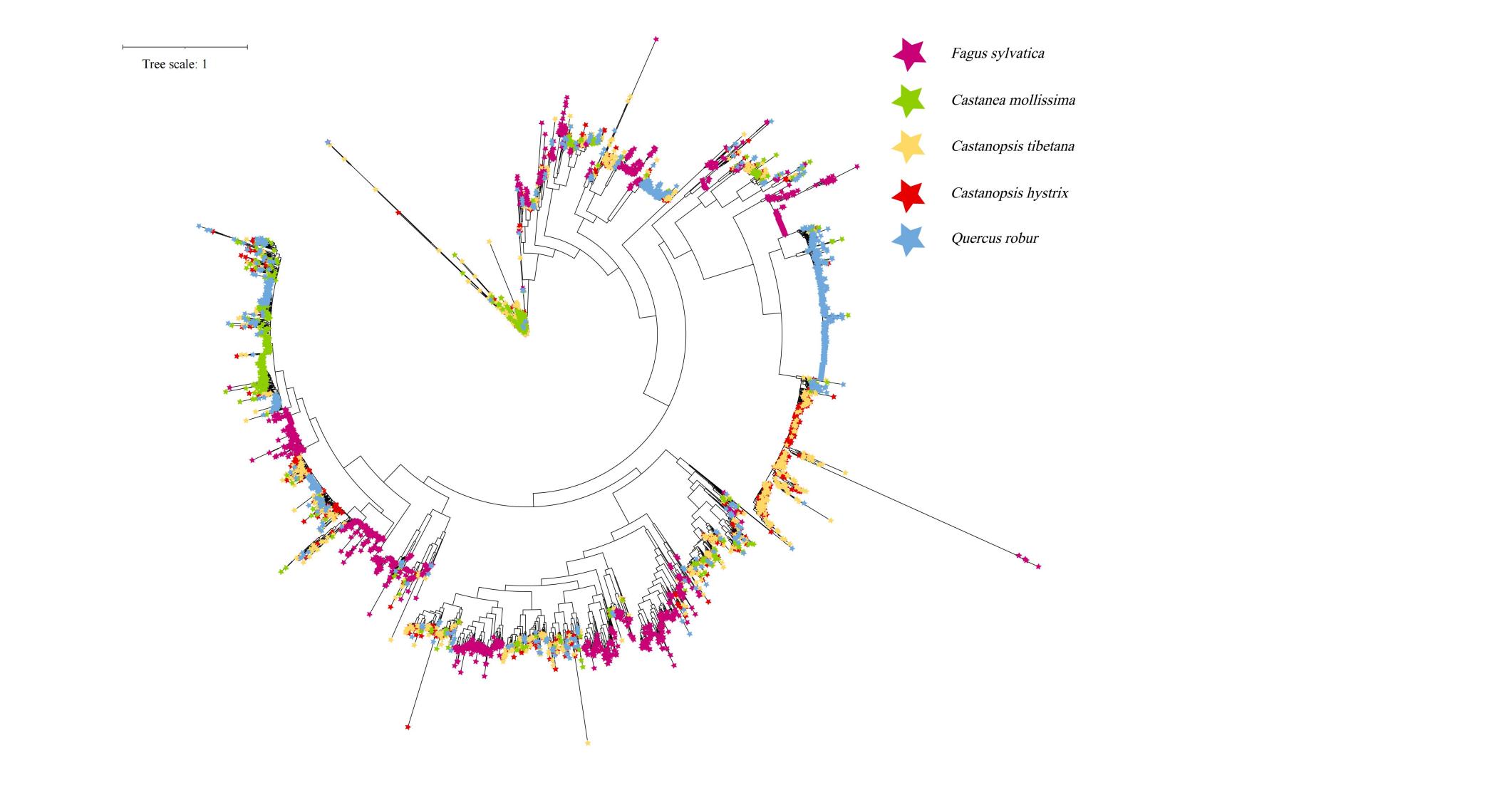

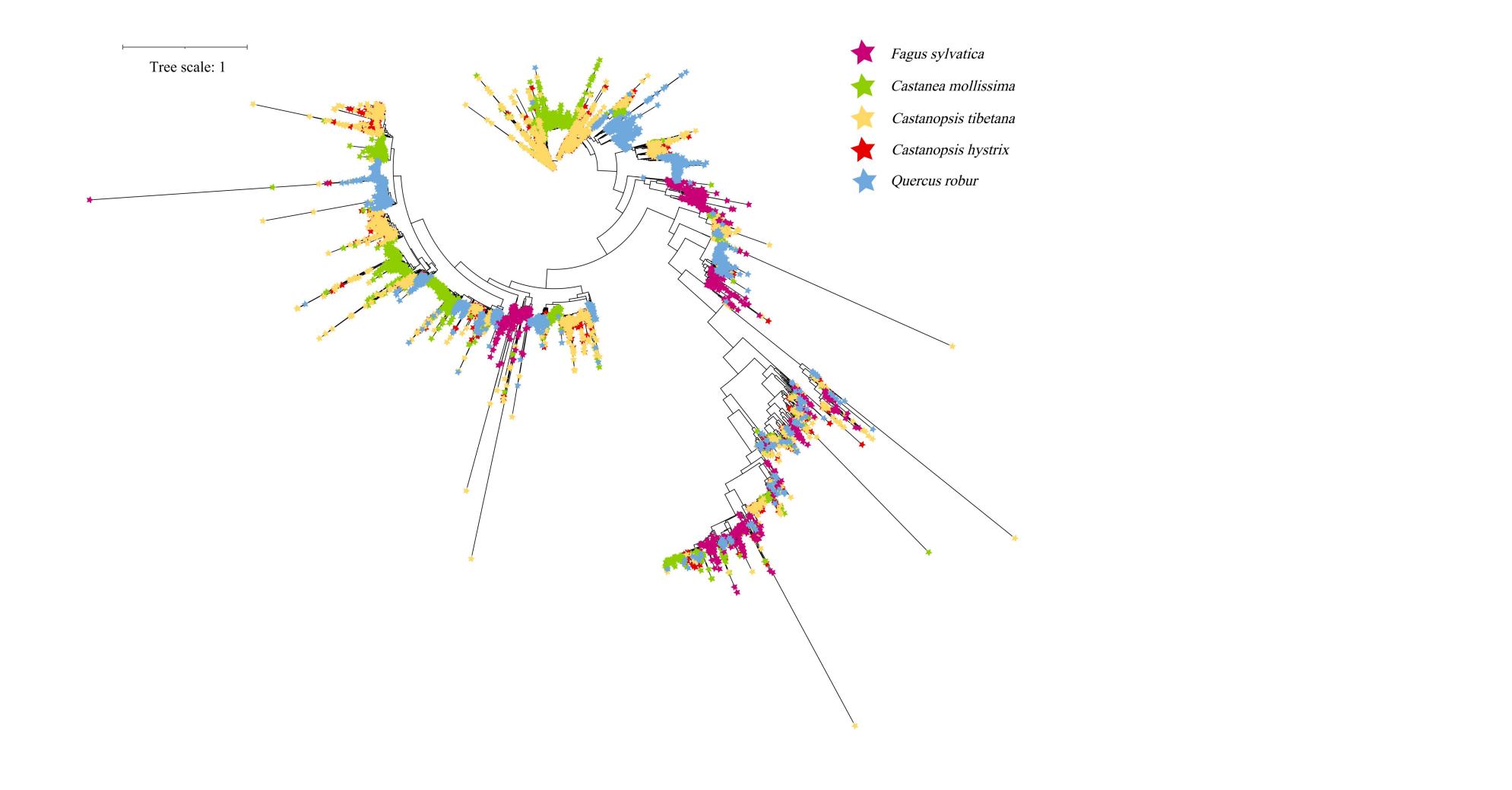


**a**

**b**

**Supplementary Figure 5.** Neighbor-joining trees of 4907 full-length Copis-type LTRs (a) and 9564 full-length Gypsy-type LTRs identified in five Fagaceae species.

**
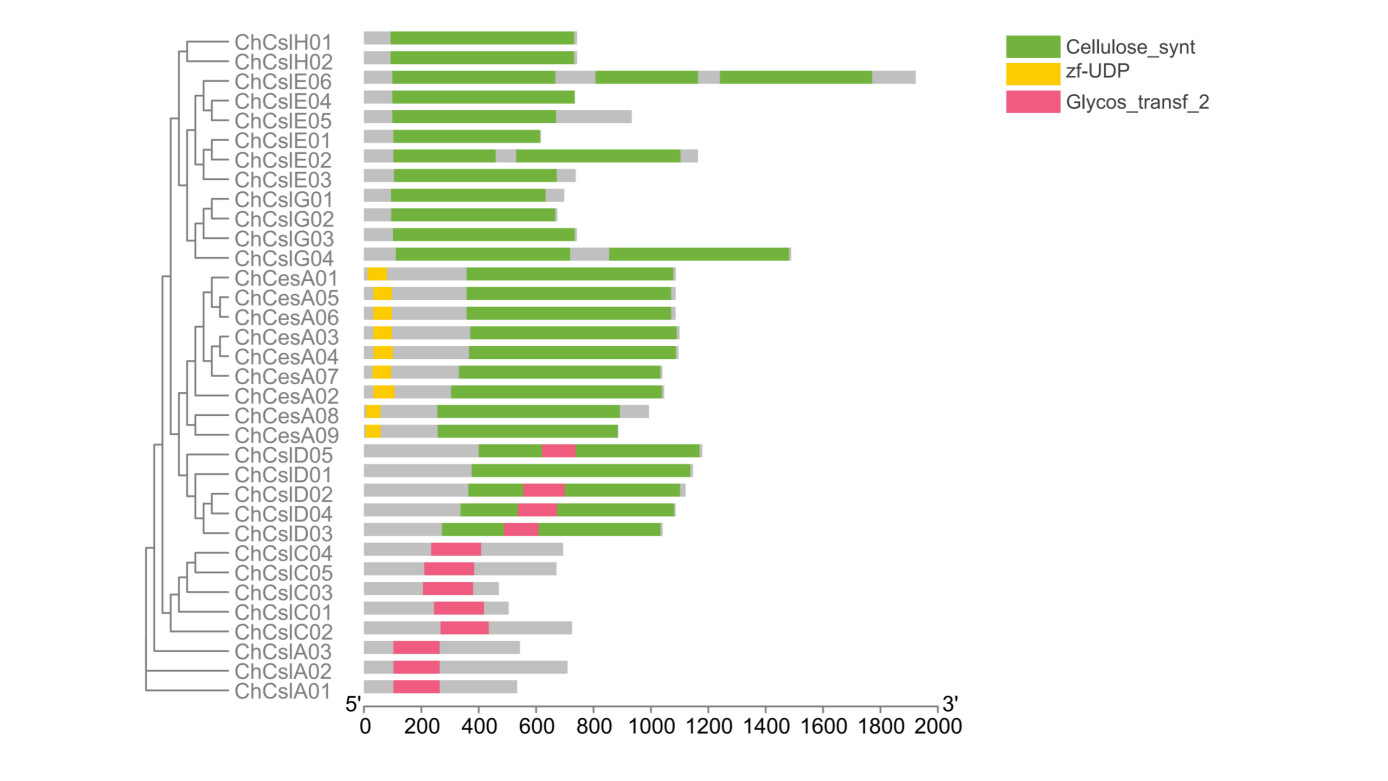
**

**Supplementary Figure 6.** The conserved protein domain compositions of 34 CesA genes in *C. hystrix*.


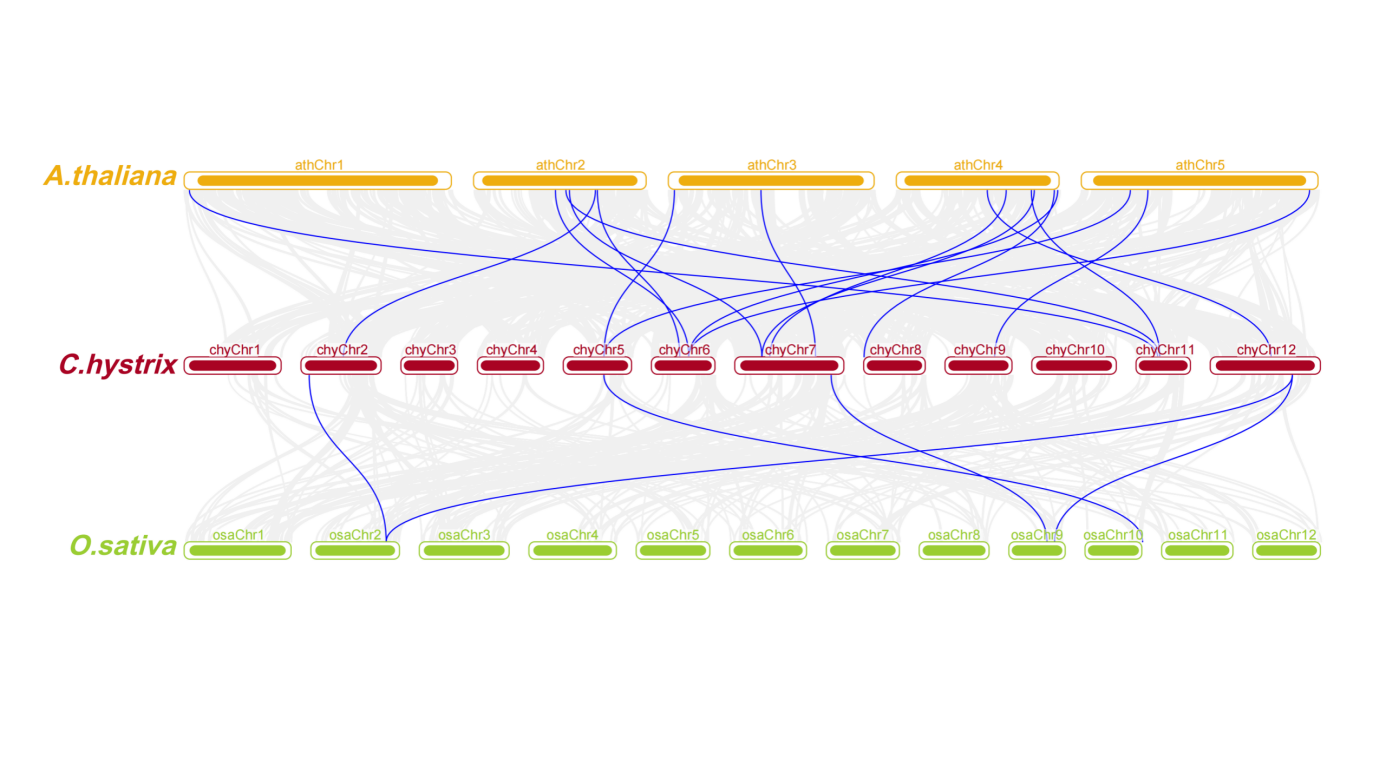


**Supplementary Figure 7.** Synteny analysis of CesA genes between *C. hystrix*, *Arabidopsis thaliana* and *Oryza sativa*. Gray lines in the background indicate the collinear blocks between genomes, while the blue lines highlight the syntenic CesA gene pairs.

**
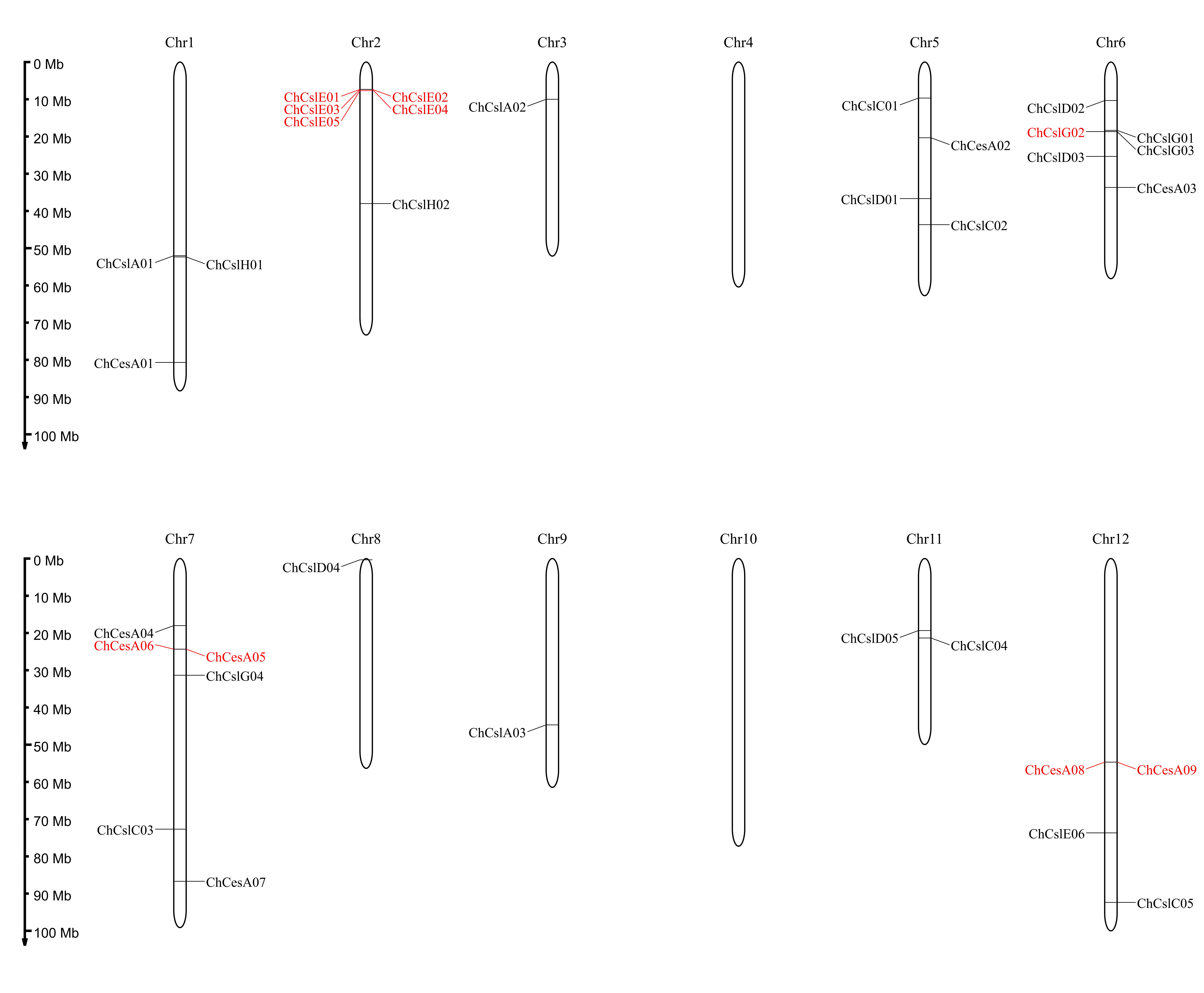
**

**Supplementary Figure 8.** Chromosomal location of CesA genes in the *C. hystrix* genome. A total of 34 CesA genes were mapped to 12 pseudo-chromosomes. Tandemly duplicated genes are indicated in red.
